# Feasibility of High-Intensity Interval Exercise in Young Adult Survivors of Pediatric Brain Tumor and Young Adults With Isocitrate Dehydrogenase–Mutant Gliomas (HYPE): Protocol for a Randomized Controlled Trial

**DOI:** 10.2196/88718

**Published:** 2026-10-15

**Authors:** Elizabeth Cox, Meghan Tahbaz, Ellaney Matarese, Rebekah L Wilson, L Nicolas Gonzalez Castro, Hajime Uno, Tabitha Cooney, Jordan Lane, Mary K Norris, Kira Bona, Rahela Aziz-Bose, Christina M Dieli-Conwright

**Affiliations:** 1 Department of Medicine, Harvard Medical School, Boston, MA, United States; 2 Department of Neurology, Cedars-Sinai Medical Center, Los Angeles, CA, United States; 3 Department of Neuroscience, Harvard University, Cambridge, MA, United States; 4 Department of Neurology, Brigham and Women’s Hospital/Mass General Brigham, Boston, MA, United States; 5 Center for Neuro-Oncology, Dana-Farber Cancer Institute, Boston, MA, United States; 6 Dana-Farber/Boston Children’s Cancer and Blood Disorders Center, Boston, MA, United States; 7 Department of Pediatrics, Harvard Medical School, Boston, MA, United States; 8 Department of Medical Oncology, Dana-Farber Cancer Institute, 450 Brookline Avenue, Boston, MA, 02215, United States, 1 617-582-8321

**Keywords:** high-intensity interval training, HIIT, pediatric brain tumors, isocitrate dehydrogenase-mutant glioma, IDH mutant glioma, AYA, exercise oncology, cognitive function

## Abstract

**Background:**

Young adult survivors of pediatric brain tumors and young adult patients with isocitrate dehydrogenase (IDH) mutant gliomas are at risk for cognitive impairment, reduced physical fitness and function, as well as poor health-related quality of life (HRQOL) and psychosocial health. Exercise improves these outcomes; however, both populations remain understudied with respect to exercise interventions. High-intensity interval training (HIIT) is a promising intervention, as it is time-efficient, safe, tolerable, enhances cognition in healthy young adults, and improves physical fitness and quality of life in adult patients and survivors with cancer. However, the feasibility of HIIT is not yet understood in survivors of pediatric brain tumors and patients with IDH-mutant glioma.

**Objective:**

The HYPE study is a pilot randomized controlled trial that aims to (1) determine the feasibility of a HIIT exercise intervention for young adult survivors of pediatric brain tumors and patients with IDH-mutant glioma, (2) determine the preliminary efficacy of HIIT to improve physical fitness and function, cognition, HRQOL, and psychosocial health, and (3) explore exercise sustainability and the impact of social determinants of health on study outcomes.

**Methods:**

A total of 30 young adult survivors of pediatric brain tumors and patients with IDH-mutant glioma (18‐45 years of age) will be randomized to an exercise or control group. The exercise group performs virtually supervised HIIT 3 times per week for 16 weeks, followed by 16 weeks of self-directed exercise. Exercise involves indoor cycling, and sessions include 20 minutes of high- and low-intensity intervals (high intensity=60-s 60%‐90% peak power output; low intensity=60-s 10% peak power output), and a 5-minute warm-up and cool-down. The control group performs a 16-week stretching program. Feasibility of the HIIT intervention is measured by the number of exercise sessions completed, including total minutes and intensity, and barriers to exercise adherence. Cognition, physical fitness and function, HRQOL, and psychosocial health are measured using standardized assessments and self-report questionnaires. Exercise sustainability is measured by change in study outcomes following the self-directed follow-up period, and social determinants of health are assessed using a self-report questionnaire.

**Results:**

Baseline data collection began in February 2024, and 13 participants have been enrolled as of June 2026. The projected study end date is December 2026. Data analysis is planned to begin in January 2027, and findings will be published in the fall of 2028.

**Conclusions:**

To our knowledge, this study is the first to use a virtually supervised HIIT intervention in young adult patients and survivors of brain tumor. The findings will elucidate the feasibility of HIIT to design and power a randomized controlled trial to address cancer- and treatment-related sequelae. They will also provide insights into the sustainability of HIIT and the impact of social determinants of health.

## Introduction

Brain and central nervous system tumors are the most prevalent solid tumors and the second most common form of pediatric cancer overall, representing 15.9% of new childhood cancer diagnoses in the United States [1]. Among young adults, primary brain tumors are also the second most common form of cancer, with an incidence of 12 per 100,000 individuals [2]; isocitrate dehydrogenase (IDH) mutant gliomas comprise the majority of these diagnoses [3]. Both populations receive treatments that can affect their neurologic function at a young age and experience prolonged survival compared to other cancer populations: survivors of pediatric brain tumor have a 5-year survival rate of 75% [4], and the majority of patients with IDH-mutant gliomas survive more than a decade [5-7]. As a result, these young adults live with long-term sequelae due to tumor and treatment. Cognitive impairment is highly prevalent in survivors of pediatric brain tumor, affecting 40%‐100% [8], and although less well-defined, it is also reported in young adults with IDH-mutant gliomas [9,10]. In both populations, impairment includes deficits in executive function, memory [11,12], and processing speed [13]. They also demonstrate reduced physical fitness and function [14-16], and limited physical activity [17-21]. Notably, cognitive and physical difficulties experienced by survivors of pediatric brain tumor can persist into young adulthood [12,22,23] and may resemble those seen in young adults with IDH-mutant gliomas. These difficulties have tangible consequences for functioning, including reduced health-related quality of life (HRQOL) and psychosocial health challenges (eg, familial and peer relationships, psychological well-being) [14,24-26]. Feasible, effective, and sustainable interventions are needed to mitigate cognitive and physical sequelae and enhance HRQOL and psychosocial health in both populations.

In survivors of pediatric brain tumor, aerobic exercise is associated with cognitive amelioration [27-29], and improvement in physical fitness, function, and HRQOL [30]. Although exercise has been studied less frequently in adults with IDH-mutant gliomas, preliminary work suggests it is safe, feasible, and may reduce tumor-related symptoms and improve HRQOL [31,32]. Commonly cited barriers include fatigue, pain [17,18], busyness, lack of gym membership [33], environmental restriction, and avoidance of traveling alone [34], as opposed to an aversion or inability to exercise [33]. Consistent with this, young adults with IDH-mutant gliomas identify exercise as an unmet lifestyle need [10]. Virtually supervised, home-based high-intensity interval training (HIIT) could directly address these barriers while providing real-time support to promote adherence and ensure safety. HIIT involves brief bouts of concentrated work alternated with recovery. It is clinically validated, safe, and accessible [35,36], and its interval structure of work and recovery is more tolerable than other forms of vigorous-intensity exercise in several clinical populations [37-40]. Further, aerobic HIIT promotes improvements in cognition [41], physical fitness [42,43], and HRQOL and psychosocial health [44-48].

Accordingly, the high-intensity interval exercise in young adult survivors of pediatric brain tumors (HYPE) study (NCT05740839) will evaluate the feasibility and preliminary efficacy of a 16-week, virtually supervised HIIT indoor cycling intervention for survivors of pediatric brain tumors who are now young adults and young adults diagnosed with IDH-mutant gliomas. The primary aim of the study is to determine feasibility, assessed through the number of exercise sessions completed, including total minutes and intensity, and barriers to exercise adherence. It is hypothesized that the intervention will be feasible for young adult survivors of pediatric brain tumors and young adults with IDH-mutant gliomas. The secondary aim is to determine the preliminary efficacy of HIIT to improve cognition, physical fitness and function, HRQOL, and psychosocial health. It is hypothesized that patients and survivors who complete the HIIT intervention will show improvements on measures of executive function, memory, processing speed, cardiorespiratory fitness, muscular strength and function, fatigue, pain, sleep, and well-being compared to baseline and a control group. Finally, tertiary aims of the study are to determine the sustainability of indoor cycling on secondary outcomes during a 16-week self-directed exercise follow-up period (ie, when supervision is removed), identify disparities in study participation, and determine the generalizability of study data to historically marginalized populations. It is hypothesized that sustainability will decrease when exercise supervision is removed, particularly among patients and survivors exposed to poverty. Associations between self-reported social determinants of health and study endpoints (feasibility and secondary outcomes) will also be explored.

## Methods

### Ethical Considerations

The HYPE study was approved by the Dana-Farber/Harvard Cancer Center Institutional Review Board (22-505). All participants provide informed consent in week 0 (Table 1) electronically or in person. Electronic consent allows participant enrollment using a distance-based approach that reduces patient burden by limiting the number of visits they are asked to make to DFCI. REDCap (Vanderbilt University) is used for storing all participant data. REDCap is a free, secure, Health Insurance Portability and Accountability Act (HIPAA) compliant web-based application. Participants are provided with equipment to perform the HIIT exercise intervention, which they keep after completing the study in lieu of compensation. The study sponsor or funders are not involved in the trial design, conduct, analysis, or reporting of the trial.

**Table 1.** SPIRIT (Standard Protocol Items: Recommendations for Interventional Trials) flow diagram for timeline of study visits.

Study time points	Week 0-1	Baseline (week 1)	Intervention (weeks 2-17)	Postintervention (week 18)	Follow-up^c^ (week 34)
Eligibility screen	✓	—^d^	—	—	—
Informed consent	✓	—	—	—	—
Allocation	—	^e^✓	—	—	—
Health questionnaire	✓	—	—		—
Medical history	✓	—	—	—	—
Exercise screening	✓	—	—	—	—
HIIT^f^ exercise intervention	—	—	✓	—	—
Exercise tolerance	—	—	✓	—	—
Attention control			✓	—	—
Primary outcome					
Feasibility	—	✓	—	✓	✓
Secondary outcomes					
Cognition	—	✓	—	✓	✓
Physical fitness and function	—	✓	—	✓	✓
Blood biomarkers	—	✓	—	✓	✓
Blood pressure	—	✓	—	✓	✓
Anthropometrics, body composition	—	✓	—	✓	✓
Health-related quality of life, psychosocial health	—	✓	—	✓	✓
Tertiary outcomes					
Exercise sustainability	—	—	—	—	✓
Demographics, social determinants of health	—	✓	—	—	—
Covariates					
Dietary assessment	—	✓	—	✓	✓
Physical activity assessment	—	✓	—	✓	✓

c Only exercise group will complete follow-up testing.

d N/A: not available.

e Allocation completed after baseline testing.

f HIIT: high-intensity interval training.

### Study Design

The study is a single-center, 2-arm pilot randomized controlled trial conducted at the Dana-Farber Cancer Institute (DFCI; Boston, Massachusetts). A total of 30 young adult survivors of pediatric brain tumors who completed tumor-directed therapy at least 2-years prior (aged 18‐45 years) and young adult patients diagnosed with IDH mutant gliomas who completed their most recent treatment (surgery, radiation, and chemotherapy) at least 1-year prior (aged 18‐45 years) will be recruited and randomly assigned to either a 16-week home-based cycling HIIT exercise intervention (exercise group; n=20) or a 16-week attention stretching control period (control group; n=10). Outcomes are measured at baseline (week 1), postintervention (week 18), and follow-up (week 34; exercise group only) (Figure 1). Participants assigned to the exercise group complete 16-weeks of HIIT exercise intervention and a 16-week self-directed exercise follow-up period. The control group performs a home-based program of the same stretches used in the exercise group. As flexibility exercises are low-intensity, low-impact, and low-volume, minimal caloric expenditure is expected to be incurred.

**Figure 1.**
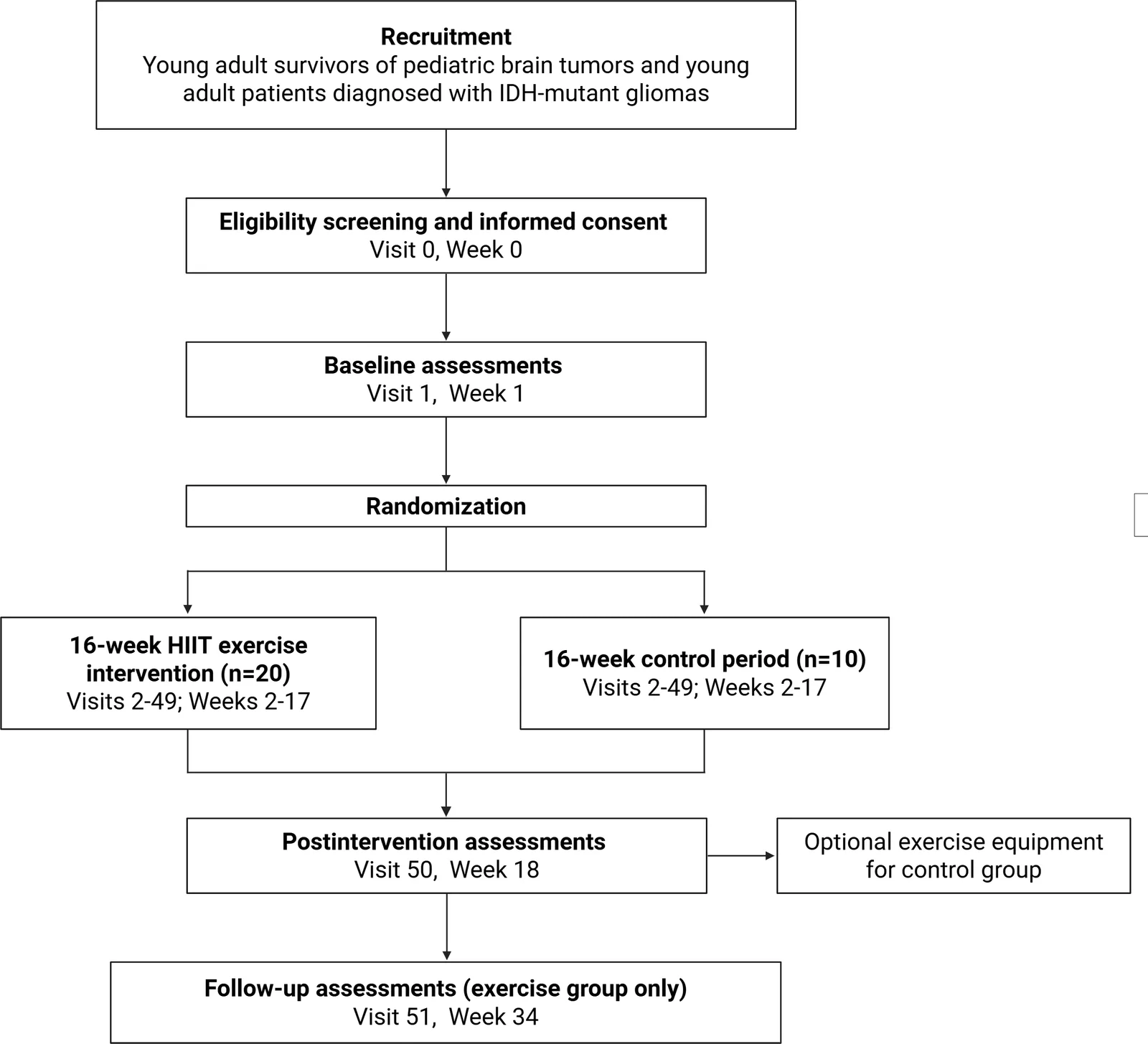
Study schema for the HYPE trial. HIIT: high-intensity interval training; IDH: isocitrate dehydrogenase.

### Participant Eligibility

Participants must meet all criteria to be eligible. Inclusion and exclusion criteria are provided in Textbox 1. Trained study staff confirm eligibility criteria and complete the protocol-specific eligibility checklist.

Textbox 1. **Inclusion and exclusion criteria.**

**Inclusion criteria**

Survivors of pediatric brain tumors:

- Histologically diagnosed brain tumor during childhood (diagnosed ≤18 years)
- 18‐45 years of age
- At least 2 years post tumor-directed therapy

Patients with isocitrate dehydrogenase (IDH)–mutant gliomas:

- Currently diagnosed with an IDH-mutant glioma (diagnosed ≥18 years)
- 18‐45 years of age
- At least 1 year since most recent surgery, radiation, or chemotherapy treatment

All survivors:

- Willing to undergo pregnancy test and use adequate contraception prior to study entry
- Able to provide informed consent prior to study-related procedures
- Able to provide physician clearance to participate in exercise program
- Speak English and/or Spanish
- Currently participates in <60 minutes of moderate or vigorous physical activity per week
- Has not smoked regularly within the previous 12 months (cigarettes or e-cigarettes). Those who infrequently smoke in social settings, and those who vape will be included
- Willing to travel to the Dana-Farber Cancer Institute

**Exclusion criteria**

All survivors:

- Diagnosis of primary spinal cord tumor
- Active malignancies
- History of chronic disease precluding participation in a high-intensity exercise program (ie, uncontrolled tumor-related epilepsy, cardiopulmonary or metabolic disease that could be exacerbated by exercise)
- Pregnancy or planning to become pregnant during study duration
- Actively on a weight loss diet

### Recruitment, Screening, and Informed Consent

Various recruitment strategies are used to identify potential participants at DFCI, including direct referral from neuro-oncologists, screening neuro-oncology clinic lists, and advertisements in patient waiting areas. Additionally, recruitment takes place outside the clinical setting via appropriate social media platforms specifically for young adult survivors of pediatric brain tumors or young adults with IDH-mutant gliomas, patient email lists, and research study advertisement websites. All interested patients and survivors complete a phone- or Zoom video call (Zoom Communications)-based screening to determine eligibility. Those who are eligible and agree to participate are asked to provide written or electronic informed consent. The use of electronic consent allows for consent to be obtained remotely. This distance-based approach can reduce participant burden and distress associated with hospital visits and reduce their number of visits to DFCI.

### Randomization and Blinding

After baseline testing, participants are randomly assigned to either the exercise or control group in an allocation ratio of 2:1 using a permuted blocked design with varying block size (with investigators blinded to block size). We use a 2:1 allocation ratio instead of 1:1 because it provides a more precise estimate for the feasibility of the exercise intervention. The study biostatistician prepared the randomization list prior to study start-up, after which a research staff member uploaded the list to REDCap. After participants have provided informed consent, study eligibility is determined, and baseline assessments are completed, a study staff member accesses REDCap to provide the randomization allocation. Given the nature of the intervention, study staff were not blinded after randomization.

### Intervention

#### Exercise Group

All exercise sessions are delivered in a home-based setting under supervision by a trained cancer exercise trainer via Zoom video call, which includes an orientation session in addition to the planned 48 exercise sessions to help get the participant familiar with the HIIT protocol and virtual exercise environment. All participants are provided with a stationary bike and are expected to engage in cycling exercise training three times weekly at home for 16 weeks; they are encouraged to complete all 48 exercise training sessions within 18 weeks (ie, allowing an extra 2 weeks to make up any missed sessions). Participants are also provided with a Wi-Fi-enabled tablet if required.

All sessions follow a standardized protocol with intensities relative to the participant’s baseline fitness level (ie, power output at VO_2_max). The cycling exercise protocol consists of a 5-minute warm-up period at 10% of maximal power output (measured by maximal cardiopulmonary fitness testing) followed by a 20-minute stimulus period, consisting of high-intensity interval bouts at 60%‐90% peak power output (PPO) (based on individual fitness level) and recovery bouts at 10% PPO. High-intensity interval and recovery bouts occur at a 1-minute:1-minute (work: recovery) ratio. Following the stimulus period, participants complete a 5-minute cool-down at 10% PPO (Figure 2). A portable heart rate monitor is used to measure heart rate prior to and during exercise. More specifically, heart rate is recorded at the beginning and end of each one-minute, high-intensity interval as well as at the end of the 1-minute recovery interval. Rate of perceived exertion and power output obtained (Watts) are recorded every minute during the stimulus period. Any deviations from the planned prescription are noted, as well as why the deviation was required. All session documents are reviewed weekly by the lead exercise trainer, with monthly meetings to discuss any participant or protocol implementation concerns.

**Figure 2.**
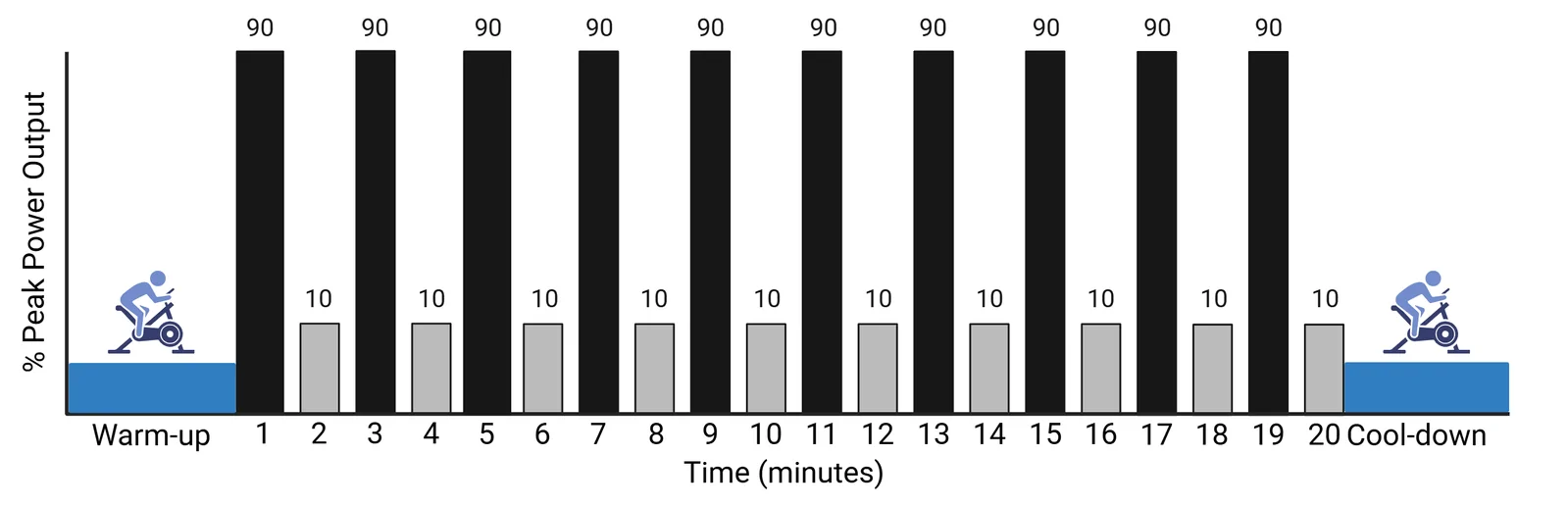
Exercise session workflow. Black bars indicate high-intensity intervals, gray bars indicate rest intervals, and blue bars indicate warm-up or cool down intervals. Exercise intensity from week 4 onwards is shown.

Exercise intensity gradually increases within the first 4 weeks of the intervention. The intensity in the first week allows for gradual familiarization with virtual supervision, while slowly introducing the body to the HIIT protocol. Each week, the “high” interval is increased by 10% until 90% PPO is reached at week 4 and then maintained at 90% PPO for the remainder of the intervention. If a participant is not able to complete the entire exercise session, the number of minutes (out of 30 min) completed during each exercise session is documented. Changes in feeling states that occur in conjunction with exercise are assessed by the Exercise-Induced Feeling Inventory [49]. This questionnaire is completed prior to exercising and repeated within 10 minutes of finishing exercise.

The safety and health of participants is of the utmost importance. At the start of each session, the trainer asks the participant if there is someone else at home, in the event that support is needed if a serious adverse event occurs. Additionally, their exercise location is confirmed in case they become unresponsive and an ambulance is required to be called. At the discretion of the trainer, a session may be terminated at any point if the participant is showing signs of or verbally informs the trainer of pain, nausea or vomiting, lightheadedness, chest pain, or loss of vision. However, this symptom list is not exhaustive. Furthermore, seizures are a common long-term symptom of brain cancer treatment. While the inclusion criteria state that only those who have not had a seizure within the past 6 months may consent, a comprehensive medical history is discussed with the participant that includes an understanding of their seizure history and any known triggers. In the event a seizure occurs during a session, the aforementioned procedures would be followed (ie, seeking assistance from someone else who is at home, or calling an ambulance). Notably, research indicates that epilepsy patients are not more likely to experience a seizure while exercising [50-52].

While the HIIT protocol is tailored to each participant based on their baseline cardiorespiratory fitness test, at the discretion of the exercise trainer, a moderate-intensity interval training (MIIT) protocol may be used if a participant is unable to achieve compliance with the HIIT program. More specifically, if a participant is unable to reach the prescribed HIIT intensity for 3 consecutive sessions, that participant is offered the MIIT program to maintain the interval structure relevant to the primary investigatory nature of the study, but also to continue to provide exercise programming for participants who may be physically unable or unwilling to participate or comply fully with a HIIT program. A single session is considered noncompliant if the number of high-intensity minutes achieving the target Watts is <70%. For example, if the participant only reached their target Watts (150W) 6 out of the 10 high-intensity minutes, this session is considered noncompliant. If this is repeated over the next 2 consecutive sessions, the participant is transitioned to the MIIT protocol. A single interval is considered noncompliant if the participant cannot stay within ±5W of the target power output for ≥40 seconds of the 1-minute interval. The intensity of the moderate interval is selected according to the highest tolerable intensity that the participant may tolerate or complete. Participants who receive MIIT and adhere to the program will be considered compliant in the feasibility analysis.

Participants in the exercise group are followed for 16 additional weeks postcompletion of the structured exercise intervention. They are encouraged to continue exercising but exercise supervision is removed (eg, self-directed exercise). Participants are provided with information pertaining to the exercise intervention and power output targets completed and keep the stationary exercise bike and heart rate monitor.

#### Control Group

During the initial 16-week period, control participants are encouraged to continue with their usual activities of daily living and complete a stretching program 3 days per week. Two upper body and 2 lower body stretches held for 30 seconds each are prescribed. Participants are provided with an instructional booklet of the stretches and a physical activity log to keep track of their activities. At the end of study participation (ie, after postintervention testing), participants in the control group are offered a self-guided 16-week HIIT exercise intervention including an optional orientation session and up to 2 supervised sessions.

### Measurements

Testing is completed at baseline (week 1), postintervention (week 18), and follow-up (week 34). Study staff members trained in exercise oncology and administration of neuropsychological assessments supervise and lead all testing, unless otherwise specified below (Table 1).

#### Primary Outcome: Feasibility

Feasibility is assessed based on intervention completion, including the number of exercise sessions completed and total number of minutes completed at the prescribed intensity. The intervention will be considered feasible if: (1) the proportion of exercise sessions attended is greater than or equal to 80%, and (2) the proportion of exercise minutes completed at the prescribed intensity is greater than or equal to 80%. Exercise adherence will be assessed using (1) the percentage and number of prescribed sessions attended out of 48 total sessions; (2) the average number of minutes of prescribed exercise completed per week out of 90 total minutes per week; and (3) the number of compliant high- or moderate-intensity intervals, defined as obtaining ±5W of target power output for ≥40 seconds of high- or moderate-intensity minute. In addition, barriers to recruitment and exercise adherence are assessed using the 17-item Barriers to Research Participation Questionnaire [53], and participant burden is assessed using the Perceived Research Burden Assessment [54]. Together, these outcomes will be used to evaluate the ability to deliver remotely supervised exercise, protocol fidelity, and intervention acceptability. Screening rate, enrollment, eligibility rate, retention rate, and occurrence of adverse events will also be assessed for future trial planning.

#### Secondary Outcomes

##### Cognition

Cognition is assessed using the National Institutes of Health (NIH) Toolbox and Montreal Cognitive Assessment (MoCA). The NIH Toolbox is a validated digital neuropsychological assessment tool used to measure various cognitive domains [55]. The following NIH Toolbox tests are administered to assess executive function, memory, and processing speed: Flanker Inhibitory Control and Attention Test to assess executive function and attention, Dimensional Change Card Sort Test to assess executive function, List Sorting Test to assess working memory (a component of executive function), Auditory Verbal Learning Test to assess immediate recall (memory), Picture Sequence Memory Test to assess episodic memory, and Oral Symbol Digit Test and Pattern Comparison Test to assess processing speed. The MoCA is a validated neuropsychological assessment used to screen for cognitive impairment, and includes memory tasks, logical reasoning, and visual reproductions [56]. An episodic memory composite is also derived from tests supported by the hippocampus and surrounding medial temporal lobe [57].

##### Physical Fitness and Function

Physical fitness and function are assessed through the sit-to-stand test, the Margaria stair climb, hand grip strength, muscular strength, and cardiorespiratory fitness. The sit-to-stand test requires participants to sit on a chair with hands placed on the opposite shoulders, crossed at the wrists. Participants will be instructed to rise to a full standing position and then sit back down again, repeated for 30 seconds. One attempt will be given, and the total number of repetitions will be recorded [58]. The Margaria stair climb is completed in a stairwell of 10 stairs. The participant climbs a flight of 10 stairs one step at a time. The time (seconds) the participant takes from stair 3 to 9 is recorded. Participants are given 3 attempts, after a familiarization practice, and the fastest time of the 3 attempts is used to calculate power (kg) [59]. Hand grip strength is measured using a hand-held dynamometer on the participant’s dominant hand (Camry Digital Hand Dynamometer). The participant is asked to complete 3 maximal grip strength attempts, and an average is calculated. Muscular strength is measured via the 1RM (repetition maximum), which is estimated from 10RM muscle strength tests on: (1) leg press, (2) leg extension, (3) leg curl (SportsArt, Mukilteo, WA & Matrix Fitness). 1RM values are calculated and reported using validated equations [60,61]. Following 1-2 warm-up sets, up to 5 attempts are given to reach the final 10RM load with a 1‐2-minute rest period between attempts. Cardiorespiratory fitness will be assessed as VO_2peak_ via expired gas analysis (Parvo Medics TrueOne 2400) using a maximal ramp (or submaximal if equipment is unavailable) cycle exercise stress test starting at 15W and increasing by 15W each minute. VO_2peak_ is reported in both relative (ml·kg^-1^·min^-1^) and absolute (L/min) terms. Maximal power output from the cardiorespiratory fitness test is also used to calculate individualized exercise prescription intensities for the HIIT program.

##### Blood Biomarkers

Serum and fasting (12 h fast) venous blood samples will be obtained by a licensed phlebotomist. Samples will be centrifuged and then aliquoted and stored at −80 degree C for future measures. Blood samples will be batch-tested at the end of the study to analyze biomarkers relating to metabolic (ie, glucose and insulin), inflammatory (ie, TNF-alpha, IL-6,8,10, and C-reactive protein), neurologic function (ie, BDNF), and stress (ie, cortisol) outcomes will take place.

##### Anthropometrics and Body Composition

A constant-tension tape measure is used to obtain waist circumference (ie, the distance around the waist using the umbilicus as the reference point) and hip circumference (ie, the distance around the widest girth of the buttocks using the greater trochanter as a landmark). Body composition is assessed via bioelectrical impedance analysis (BIA) using a validated device (Tanita 780, Arlington Heights, IL). Participants are asked to remove their shoes and socks after which height is taken using a stadiometer. They then proceed to the BIA device and stand still while holding the handles with their hands at their sides. The device estimates body fat using an algorithm based on their age, sex, height, and body weight.

##### HRQOL and Psychosocial Health

HRQOL and psychosocial health are assessed using the Functional Assessment of Cancer Therapy-General (FACT-G), Brief Fatigue Inventory (BFI), and the Brief Pain Inventory (BPI). The FACT-G is a general questionnaire comprised of 27 items validated to measure physical, emotional, social, and functional well-being of patients and survivors with cancer [62]. The BFI is used to rapidly assess the severity and impact of cancer-related fatigue including 6 items that correlate with QOL measures [63]. The BPI is used to assess the impact of pain on participants’ QOL, and includes a sensory and reactive dimension and has been previously validated in survivors of cancer [64]. Sleep quality is assessed using the Pittsburgh Sleep Quality Index, which contains 19 questions evaluating 7 domains of sleep: subjective sleep quality, sleep latency, sleep duration, habitual sleep efficiency, sleep disturbances, use of sleep medication, and daytime dysfunction [65,66].

To address participant burden, fatigue, and cognitive impairment, assessments are scheduled at times that are convenient for participants, and whenever possible, coordinated with existing clinical visits to reduce travel and visits to DFCI. If needed, assessments may be completed over 2 days. The order of assessments is standardized so that potential effects of fatigue, as well as acute exercise from the cardiorespiratory fitness test on cognitive performance [41], occur uniformly across participants.

### Tertiary Outcomes

#### Exercise Sustainability

The sustainability of exercise when supervision is removed is measured through weekly physical activity logs completed by the exercise group during the 16-week self-directed exercise follow-up period. The logs can be completed electronically via REDCap, MyCap mobile app, or on paper.

#### Social Determinants of Health Disparities

To identify disparities in study participation and determine the generalizability of study data to historically marginalized populations, self-reported sociodemographic information and social determinants of health are collected at baseline using the brief Household Survey. This measure is collected within a 2-week window from study consent. The Household Survey intends to gather information about the socioeconomic status of the participant’s primary household and can be completed by the participant or with the assistance of an identified caregiver as appropriate. The Household Survey is available in English and Spanish and includes self-reported health insurance, primary language, ethnicity, race, marital status, education, health literacy, income, household size, and measures of household material hardship (food, housing, utility, or transportation insecurity) [67].

### Covariates

Participants additionally undergo dietary and physical activity assessments. An automated, self-administered 24-hour dietary assessment tool is used to assess recent dietary patterns for 2 weekdays and 1 weekend day (nonconsecutive where possible) [68]. To measure 7-day physical activity, participants are provided with a hip-worn accelerometer (ActiGraph, Pensacola, FL) to wear for 7 days at baseline, postintervention, and follow-up (exercise group only) testing visits and are instructed to wear the water-resistant accelerometer except when sleeping, swimming, or bathing. The device records activity data and is electronically transferred to a computer via USB cable at the time of completion.

### Adverse Events

Individuals are asked about adverse events at each supervised visit and are provided with a phone number to report potential intervention-related adverse events to study staff. All adverse events are reported, and these are evaluated for the likelihood that they occurred as a result of the intervention (very likely to be related, likely to be related, possibly related, or unlikely to be related). Adverse events are reported to the principal investigator and project manager as soon as study staff learns of their occurrence, as well as to the treating neuro-oncologist if deemed necessary, such as in the event of a seizure or acute worsening of neurologic function; this includes events that occur after the initiation of the study intervention, during the intervention, and within 30 days of intervention completion. Additionally, participants are instructed to report any serious poststudy events that might reasonably be related to participation in this study. Serious adverse events must be reported within 24 hours of learning of the event. The principal investigator reports adverse events that are severe, life-threatening or disabling, and fatal, and are possibly, probably, or definitely related to the intervention to the institutional review board. All other adverse events are logged and documented.

All adverse events, both serious and nonserious, and deaths that are encountered from initiation of the study intervention through 30 days of intervention completion are followed to their resolution, or until the participating investigator assesses the participant to be stable, or determines the event to be irreversible, or the participant is lost to follow-up. The presence and resolution (if applicable) of adverse events is documented and recorded in the participant’s regulatory folder.

### Data Monitoring and Management

The study team will collect, manage, and perform quality checks on the data for this study (eg, verification of REDCap data entries). All data is monitored for timeliness of submission, completeness, and adherence to protocol requirements. Monitoring begins at the time of participant registration and continues during protocol performance and completion. Potential audits or inspections may be conducted by representatives designated by the principal investigator or Dana-Farber/Harvard Cancer Center. REDCap is used for storing participant outcome data, and the study database is password-protected and stored on a secure network.

### Sample Size Calculation

We designed the study to have a total of 25 complete cases for the analyses. Considering a possible dropout rate of 15%, the total accrual goal is 30 participants. This sample size was determined based on recommendations of sample sizes between 24 and 50 for pilot studies assessing the feasibility of exercise interventions and estimating effect sizes for designing future studies [69-71]. The primary endpoint of this study is the feasibility of the HIIT exercise intervention. With the 2:1 allocation, 20 participants are allocated to the exercise group. We are able to observe an 80% (16/20) or higher proportion of minutes of intended prescribed exercise completed with a probability of 80%. The study will be able to detect an intervention effect between groups with 80% power, at a 0.05 2-sided α level, when the effect size (Cohen *d*) is 1.25 or higher. Given the exploratory nature of the study, no adjustment for multiple comparisons will be applied.

### Statistical Analysis

To assess feasibility endpoints, the analysis will include all participants who are assigned to the exercise intervention, including those who never start the exercise intervention. The primary analysis population includes all eligible participants who complete the baseline (week 1) and postintervention (week 18) assessments. The secondary analysis population includes eligible participants who complete all assessments up to Week 34. The primary analysis will potentially include missing values due to population dropout, and the all available case analysis method will be used. Multiple imputations will be used for sensitivity analyses. Analyses stratified by subpopulation will also be conducted.

To test hypotheses related to the feasibility of a 16-week virtually supervised HIIT exercise intervention for young adult survivors of pediatric brain tumors and young adults with IDH-mutant gliomas (primary objective), the proportion of participants who attend 70% of the total prescribed number of exercise sessions and complete 70% of the total prescribed minutes per exercise session will be calculated; a corresponding 0.95 exact CI will also be calculated. To test hypotheses related to the intervention effects on change in cognitive-, physical-, HRQOL-, and psychosocial health–related outcomes from baseline to week 18 (secondary objective), within- and between-group comparisons will be performed on the primary analysis population. Paired tests will be used to assess within-group differences, and 2-sample *t* tests will be used to assess differences between groups for each outcome; mean difference scores and a corresponding 0.95 CI will be calculated and normalizing transformations will be applied as needed. Analysis of covariance (ANCOVA) will also be used to assess mean differences in each outcome while adjusting for participant baseline characteristics and clinical covariates. Chi-square tests will be used for between-group comparison of binary outcomes (eg, occurrence of adverse events). The magnitude of the intervention effect on binary outcomes will be summarized by odds ratio and corresponding 0.95 CI. For each analysis, a 2-sided *P* value ≤.05 will be considered statistically significant. Because this is a pilot study, we will not perform corrections for multiple comparisons. To test hypotheses related to the sustainable effects of the intervention and associations between outcome measures (tertiary objective), the difference between scores at follow-up (week 34) and postintervention (week 18) will be assessed in the secondary analysis population. Mean and median difference scores and corresponding 0.95 CIs will be calculated, and paired *t* tests will be performed. Wilcoxon signed-rank tests will be used if model assumptions are violated. Linear mixed-effects models will be applied to each outcome’s longitudinal data (week 1, 18, and 34) to explore trends over time and the association between outcome and baseline characteristics. Associations between outcome measures will be assessed using Pearson correlation, or Spearman rank correlation if model assumptions are violated. Additional statistics related to future trial planning will include screening rate, enrollment, eligibility rate, retention rate, and occurrence of adverse events.

For the exploratory health equity aim, participant characteristics will be described by self-reported race, ethnicity, and household material hardship exposure as compared to US epidemiologic data to evaluate participant representativeness. Associations will be explored between self-reported social determinants of health, using household material hardship as the primary exposure, and study endpoints, including (1) feasibility measures, (2) baseline and end-intervention cognitive status, and HRQOL.

## Results

This study was funded in October 2022. Baseline data collection began in February 2024, and 13 participants have been enrolled as of June 2026. The projected study end date is December 2026. Data analysis is planned to begin in January 2027. Primary outcomes will be analyzed first, followed by secondary and tertiary outcomes. Findings will be published in the fall of 2028 in the order of analysis.

## Discussion

### Anticipated Findings

The HYPE study advances exercise oncology by evaluating the feasibility of HIIT in 2 understudied populations and providing preliminary insights into its impact on cancer-related sequelae and exercise sustainability. It is hypothesized that ≥80% of young adult survivors of pediatric brain tumors and young adults with IDH-mutant gliomas assigned to the exercise group will attend ≥70% of prescribed HIIT exercise sessions and complete ≥70% of prescribed minutes per exercise session. HIIT will also improve cognitive, physical, HRQOL, and psychosocial health outcomes relative to the control group and baseline. Additionally, it is hypothesized that the removal of exercise supervision after the intervention period will attenuate intervention effects at follow-up and that poverty exposure will moderate this effect.

### Comparison With Prior Work

The safety, tolerability, and efficiency of HIIT position it as a feasible intervention in young adult survivors of pediatric brain tumors and young adults with IDH-mutant gliomas. However, despite these advantages and the increased use of aerobic exercise to improve cancer-related outcomes, most exercise oncology interventions to date have used moderate-intensity continuous training [72]. HIIT may offer a particularly time-efficient alternative, as lower exercise volumes are needed to achieve greater improvements in cardiovascular and physical fitness outcomes, including VO_2_max [73]. This efficiency is especially important given the physical activity barriers faced by young adult survivors of pediatric brain tumors and young adults with IDH-mutant gliomas. Further, HIIT may also elicit neurophysiological benefits relevant to cognition. Compared to moderate-intensity continuous training, HIIT elicits greater oxygen use [74] and cerebral oxygenation [75], which may contribute to stronger cognitive effects. Consistent with this possibility, HIIT has been shown to promote greater cognitive improvements in healthy young adults [76] and survivors of cancer [77], and higher aerobic exercise intensity has been associated with structural brain changes that support efficient cognitive processing [78]. Finally, HIIT is often perceived as more enjoyable than continuous exercise [79], which may further support physical activity engagement and feasibility of the HYPE study [80-82].

### Secondary Findings

While the effects of HIIT on cognition in young adult survivors of pediatric brain tumors and young adults with IDH-mutant gliomas are currently unknown, HYPE aims to fill this knowledge gap. Executive function, memory, and processing speed are sensitive to the benefits of chronic aerobic HIIT (>8 weeks) in healthy adults [41], and to aerobic exercise more broadly in both survivors of pediatric brain tumors [27,28] and adults with grade 2/3 gliomas [31]. Mechanistically, exercise-related improvements likely reflect neurobiological effects in brain networks that support these cognitive domains [27-29,83-85]. Given the vulnerability of survivors of pediatric brain tumors to tumor- and treatment-related brain disruption (eg, tumor location, age at diagnosis, cranial radiation, and chemotherapy) [12,86] and similar therapy-related cognitive risks in young adults with IDH-mutant gliomas (eg, tumor progression, radiation, and chemotherapy) [9,87,88], it is plausible that HIIT will produce measurable cognitive improvements in both populations. Future research should investigate the neurobiological mechanisms that may drive HIIT-cognitive improvement in these populations as well.

HYPE is also the first study to investigate the effects of HIIT on cardiorespiratory fitness in survivors of pediatric brain tumors and patients with IDH-mutant gliomas. Like cognitive impairment, reduced cardiorespiratory fitness is often due to tumor- and treatment-related effects, such as endocrine and cardiovascular conditions [89,90]; in young adult survivors of pediatric brain tumors, levels can resemble those of adults 60 years and older [14], and lower cardiovascular fitness in patients with IDH-mutant gliomas is associated with fatigue that interferes with daily functioning [16]. Evidence from adult patients and survivors with cancer shows that HIIT safely improves cardiorespiratory fitness [42,43], and aerobic exercise yields similar benefits in survivors of pediatric brain tumors [91] and young adults with stable grade 2/3 gliomas [92]. Benefits of aerobic exercise also extend beyond fitness, including improved physical functioning [93], higher self-reported physical activity, and lower BMI [82]. Accordingly, we expect HIIT to improve physical fitness and functioning, and related outcomes in young adult survivors of pediatric brain tumors and young adults with IDH-mutant gliomas. Additionally, given their high symptom burden [15,16], routine assessment of cardiorespiratory fitness is important. The HYPE study is also the first to assess patients’ and survivors’ cardiorespiratory fitness over a 32-week period (16-week intervention plus 16-week follow-up).

Psychosocial health is a key component of HRQOL that may also improve with HIIT. Survivors of pediatric brain tumors report the poorest HRQOL among survivors of pediatric cancer [94], with 60% not achieving complete independence in adulthood—a limitation that is in part driven by reduced aerobic capacity and adaptive physical functioning [95]. Cognitive impairment is also associated with poor social adjustment [96-98] and mediates social outcomes [96] in these survivors. Young adults with IDH-mutant gliomas similarly report unmet psychosocial needs [10]. Aerobic exercise improves psychosocial health in adults with grade 2/3 gliomas [31] and vigorous exercise promotes similar improvement in adult survivors of childhood cancer [44]. By reducing anxiety, depression, and stress [99], HIIT may help counteract emotional effects related to cancer management and survivorship [45-48].

### Tertiary Findings

Equity considerations are integral to the HYPE study. Poverty and adverse social determinants of health negatively affect cognitive [100,101] and physical [102] outcomes and represent modifiable risk factors for late effects of brain tumors and treatment. Given the risk of cognitive and physical sequelae in young adult survivors of pediatric brain tumors and young adults with IDH-mutant gliomas, and the benefits of exercise, an equity-centered study design (eg, remote consent, provision of exercise equipment, language accessibility), and collection of self-reported sociodemographic and social determinants of health data are necessary to assess the generalizability and sustainability of the HIIT intervention.

### Strengths and Limitations

The strengths of this study include a 2-arm randomized controlled design, access to a large patient population, and the comprehensive measures used to determine feasibility of the HIIT intervention, its preliminary efficacy to improve cognitive, physical, HRQOL, and psychosocial health, and exercise sustainability. Limitations of the study also merit consideration. First, as a single-center study that operates within the context of a comprehensive cancer treatment and research institution, there is the potential for recruitment bias. We have attempted to mitigate this bias through recruiting via social media platforms for young adult survivors of pediatric brain tumors and patients with IDH-mutant gliomas, and research study advertisement websites, rather than solely in clinical settings. Of note, study recruitment is slow due to the rarity of the disease and time commitment. Accrual will be monitored and the need for future multisite trials will be assessed. Additionally, participants may already have an established interest in sports or other physical activities, which could influence their baseline fitness level. To address this, physical activity at baseline is captured by an accelerometer over a 7-day period and the health questionnaire, allowing analyses to account for baseline fitness. In light of the study’s feasibility aims, the control group was also not matched for attention exposure to that of the exercise group. Future efficacy trials should use equivalent attention exposures between groups. Finally, given follow-up testing is limited to participants in the exercise group, sustainability analyses will exclusively assess within-group change. While the study cannot establish comparative sustainability with the control group, evaluating sustainability of no-exercise is outside of its scope.

### Conclusions

In summary, the HYPE study will evaluate the feasibility of virtually supervised HIIT, generate preliminary evidence on its efficacy to improve cancer-related sequelae, and explore exercise sustainability in young adult survivors of pediatric brain tumors and young adults with IDH-mutant gliomas. The findings of this pilot feasibility randomized controlled trial will inform the design of a larger efficacy trial.

## Supplementary material

10.2196/88718 Checklist 1 SPIRIT checklist.

## Abbreviations

ANCOVA: analysis of covariance
BFI: Brief Fatigue Inventory
BIA: bioelectrical impedance analysis
BPI: Brief Pain Inventory
DFCI: Dana-Farber Cancer Institute
FACT-G: Functional Assessment of Cancer Therapy-General
HIIT: high-intensity interval training
HIPAA: Health Insurance Portability and Accountability Act
HRQOL: health-related quality of life
HYPE: high-intensity interval exercise in young adult survivors of pediatric brain tumors
IDH: isocitrate dehydrogenase
MIIT: Moderate-intensity Interval Training
MoCA: Montreal Cognitive Assessment
NIH: National Institutes of Health
PPO: peak power output

## References

[1] Cancer stat facts: childhood brain and other nervous system cancer (ages 0–19). SEER Cancer Statistics Review.

[2] Price M, Ballard C, Benedetti J (2024). CBTRUS statistical report: primary brain and other central nervous system tumors diagnosed in the United States in 2017-2021. Neuro Oncol.

[3] Miller JJ, Gonzalez Castro LN, McBrayer S (2023). Isocitrate dehydrogenase (IDH) mutant gliomas: a Society for Neuro-Oncology (SNO) consensus review on diagnosis, management, and future directions. Neuro Oncol.

[4] Taylor AJ, Little MP, Winter DL (2010). Population-based risks of CNS tumors in survivors of childhood cancer: the British childhood cancer survivor study. J Clin Oncol.

[5] Olar A, Wani KM, Alfaro-Munoz KD (2015). IDH mutation status and role of WHO grade and mitotic index in overall survival in grade II-III diffuse gliomas. Acta Neuropathol.

[6] van den Bent MJ, French PJ, Brat D (2024). The biological significance of tumor grade, age, enhancement, and extent of resection in IDH-mutant gliomas: how should they inform treatment decisions in the era of IDH inhibitors?. Neuro Oncol.

[7] Lanman TA, Gonzalez Castro LN (2025). Targeting of mutant isocitrate dehydrogenase in glioma: a systematic review. Cancers (Basel).

[8] Palmer SL, Armstrong C, Onar-Thomas A (2013). Processing speed, attention, and working memory after treatment for medulloblastoma: an international, prospective, and longitudinal study. J Clin Oncol.

[9] Lanman TA, Gross H, Faria CM (2025). Factors associated with cognitive impairment in long-term IDH-mutant glioma survivors. J Neurooncol.

[10] Fox RG, Chukwueke UN, Sannes T (2025). Glioma resource outreach with support: a program to identify and initiate supportive care interventions for unmet needs among adult lower-grade glioma patients. Neuro-Oncol Pract.

[11] Stavinoha PL, Askins MA, Powell SK, Pillay Smiley N, Robert RS (2018). Neurocognitive and psychosocial outcomes in pediatric brain tumor survivors. Bioengineering (Basel).

[12] Oyefiade A, Paltin I, De Luca CR (2021). Cognitive risk in survivors of pediatric brain tumors. J Clin Oncol.

[13] Al Dahhan NZ, Powanwe AS, Ismail M (2024). Network connectivity underlying information processing speed in children: Application of a pediatric brain tumor survivor injury model. Neuroimage Clin.

[14] Ness KK, Hudson MM, Ginsberg JP (2009). Physical performance limitations in the childhood cancer survivor study cohort. J Clin Oncol.

[15] Wolfe KR, Hunter GR, Madan-Swain A, Reddy AT, Baños J, Kana RK (2012). Cardiorespiratory fitness in survivors of pediatric posterior fossa tumor. J Pediatr Hematol Oncol.

[16] van Coevorden-van Loon EMP, Horemans H, Heijenbrok-Kal MH (2023). Physical fitness and its association with fatigue in patients with low-grade glioma. Disabil Rehabil.

[17] Ng AH, Ngo-Huang A, Vidal M (2021). Exercise barriers and adherence to recommendations in patients with cancer. JCO Oncol Pract.

[18] Recklitis CJ, Liptak C, Footer D, Fine E, Chordas C, Manley P (2019). Prevalence and correlates of pain in adolescent and young adult survivors of pediatric brain tumors. J Adolesc Young Adult Oncol.

[19] Swensen SN, Figuracion KCF, Venur VA (2024). Treatment options for IDH-mutant malignant gliomas. Curr Treat Options Oncol.

[20] Ruden E, Reardon DA, Coan AD (2011). Exercise behavior, functional capacity, and survival in adults with malignant recurrent glioma. J Clin Oncol.

[21] Nicole Culos-Reed S, Leach HJ, Capozzi LC, Easaw J, Eves N, Millet GY (2017). Exercise preferences and associations between fitness parameters, physical activity, and quality of life in high-grade glioma patients. Support Care Cancer.

[22] Ness KK, Leisenring WM, Huang S (2009). Predictors of inactive lifestyle among adult survivors of childhood cancer: a report from the childhood cancer survivor study. Cancer.

[23] Wagner AP, Carroll C, White SR (2020). Long-term cognitive outcome in adult survivors of an early childhood posterior fossa brain tumour. Int J Clin Oncol.

[24] Reuvers MJP, Burgers VWG, Koekkoek JAF (2025). “Aiming at a moving target”-the daily life experiences of adolescents and young adults with a low-grade glioma. Neurooncol Pract.

[25] Rimmer B, Bolnykh I, Dutton L (2023). Health-related quality of life in adults with low-grade gliomas: a systematic review. Qual Life Res.

[26] Schreiber JE, Gurney JG, Palmer SL (2014). Examination of risk factors for intellectual and academic outcomes following treatment for pediatric medulloblastoma. Neuro Oncol.

[27] Riggs L, Piscione J, Laughlin S (2017). Exercise training for neural recovery in a restricted sample of pediatric brain tumor survivors: a controlled clinical trial with crossover of training versus no training. Neuro Oncol.

[28] Szulc-Lerch KU, Timmons BW, Bouffet E (2018). Repairing the brain with physical exercise: cortical thickness and brain volume increases in long-term pediatric brain tumor survivors in response to a structured exercise intervention. Neuroimage Clin.

[29] Cox E, Bells S, Timmons BW (2020). A controlled clinical crossover trial of exercise training to improve cognition and neural communication in pediatric brain tumor survivors. Clin Neurophysiol.

[30] Braam KI, van der Torre P, Takken T, Veening MA, van Dulmen-den Broeder E, Kaspers GJL (2013). Physical exercise training interventions for children and young adults during and after treatment for childhood cancer. Cochrane Database Syst Rev.

[31] Gehring K, Stuiver MM, Visser E (2020). A pilot randomized controlled trial of exercise to improve cognitive performance in patients with stable glioma: a proof of concept. Neuro Oncol.

[32] Miklja Z, Gabel N, Altshuler D, Wang L, Hervey-Jumper SL, Smith S (2022). Exercise improves health-related quality of life sleep and fatigue domains in adult high- and low-grade glioma patients. Support Care Cancer.

[33] Arroyave WD, Clipp EC, Miller PE (2008). Childhood cancer survivors’ perceived barriers to improving exercise and dietary behaviors. Oncol Nurs Forum.

[34] Brinkman TM, Li Z, Neglia JP (2013). Restricted access to the environment and quality of life in adult survivors of childhood brain tumors. J Neurooncol.

[35] Casla S, Hojman P, Márquez-Rodas I (2015). Running away from side effects: physical exercise as a complementary intervention for breast cancer patients. Clin Transl Oncol.

[36] Buffart LM, Newton RU, Chinapaw MJ (2015). The effect, moderators, and mediators of resistance and aerobic exercise on health-related quality of life in older long-term survivors of prostate cancer. Cancer.

[37] Mely L, Meurisse O, Norberciak L (2026). Feasibility and tolerance of high-intensity interval training in adults with cystic fibrosis. Arch Rehabil Res Clin Transl.

[38] Gallo PM (2021). High-intensity interval training for neurodegenerative conditions. ACSMs Health Fit J.

[39] Wu X, Yang D, Zhu Q, Xiao Y, Huang H (2025). The safety and efficacy of high-intensity interval training (HIIT) in post-stroke patients with moderate functional impairment: a systematic review and meta-analysis. Front Neurol.

[40] Adams V, Alves M, Fischer T (2015). High-intensity interval training attenuates endothelial dysfunction in a Dahl salt-sensitive rat model of heart failure with preserved ejection fraction. J Appl Physiol (1985).

[41] Liu K, Zhao W, Li C (2024). The effects of high-intensity interval training on cognitive performance: a systematic review and meta-analysis. Sci Rep.

[42] Isanejad A, Nazari S, Gharib B, Motlagh AG (2023). Comparison of the effects of high-intensity interval and moderate-intensity continuous training on inflammatory markers, cardiorespiratory fitness, and quality of life in breast cancer patients. J Sport Health Sci.

[43] Wallen MP, Hennessy D, Brown S (2020). High-intensity interval training improves cardiorespiratory fitness in cancer patients and survivors: a meta-analysis. Eur J Cancer Care (Engl).

[44] Tonorezos ES, Ford JS, Wang L (2019). Impact of exercise on psychological burden in adult survivors of childhood cancer: a report from the childhood cancer survivor study. Cancer.

[45] Cheung AT, Li WHC, Ho LLK (2019). Impact of brain tumor and its treatment on the physical and psychological well-being, and quality of life amongst pediatric brain tumor survivors. Eur J Oncol Nurs.

[46] Cormie P, Nowak AK, Chambers SK, Galvão DA, Newton RU (2015). The potential role of exercise in neuro-oncology. Front Oncol.

[47] Walbert T, Avila EK, Boele FW (2025). Symptom management in isocitrate dehydrogenase mutant glioma. Neurooncol Pract.

[48] van der Meer PB, Dirven L, Hertler C (2023). Depression and anxiety in glioma patients. Neurooncol Pract.

[49] Annesi J (2005). Relations of self-motivation, perceived physical condition, and exercise-induced changes in revitalization and exhaustion with attendance in women initiating a moderate cardiovascular exercise regimen. Women Health.

[50] Perreault S, Lober RM, Davis C, Stave C, Partap S, Fisher PG (2014). Sports and childhood brain tumors: can I play?. Neurooncol Pract.

[51] Capovilla G, Kaufman KR, Perucca E, Moshé SL, Arida RM (2016). Epilepsy, seizures, physical exercise, and sports: a report from the ILAE task force on sports and epilepsy. Epilepsia.

[52] Gates JR, Spiegel RH (1993). Epilepsy, sports and exercise. Sports Med.

[53] Kibler JL, Brisco K (2006). Evaluation of a brief questionnaire for assessing barriers to research participation. Ethn Dis.

[54] Lingler JH, Schmidt KL, Gentry AL, Hu L, Terhorst LA (2014). A new measure of research participant burden: brief report. J Empir Res Hum Res Ethics.

[55] Weintraub S, Dikmen SS, Heaton RK (2013). Cognition assessment using the NIH toolbox. Neurology.

[56] Nasreddine ZS, Phillips NA, Bédirian V (2005). The Montreal Cognitive Assessment, MoCA: a brief screening tool for mild cognitive impairment. J Am Geriatr Soc.

[57] Holland D, Brewer JB, Hagler DJ, Fennema-Notestine C, Dale AM (2009). Subregional neuroanatomical change as a biomarker for Alzheimer’s disease. Proc Natl Acad Sci U S A.

[58] Jones CJ, Rikli RE, Beam WC (1999). A 30-s chair-stand test as a measure of lower body strength in community-residing older adults. Res Q Exerc Sport.

[59] Bean JF, Kiely DK, LaRose S, Alian J, Frontera WR (2007). Is stair climb power a clinically relevant measure of leg power impairments in at-risk older adults?. Arch Phys Med Rehabil.

[60] Knutzen KM, Brilla LR, Caine D (1999). Validity of 1RM prediction equations for older adults. J Strength Cond Res.

[61] Reynolds JM, Gordon TJ, Robergs RA (2006). Prediction of one repetition maximum strength from multiple repetition maximum testing and anthropometry. J Strength Cond Res.

[62] Cella DF, Tulsky DS, Gray G (1993). The Functional Assessment of Cancer Therapy scale: development and validation of the general measure. J Clin Oncol.

[63] Mendoza TR, Wang XS, Cleeland CS (1999). The rapid assessment of fatigue severity in cancer patients: use of the Brief Fatigue Inventory. Cancer.

[64] Hølen JC, Lydersen S, Klepstad P, Loge JH, Kaasa S (2008). The brief pain inventory: pain’s interference with functions is different in cancer pain compared with noncancer chronic pain. Clin J Pain.

[65] Buysse DJ, Reynolds CF, Monk TH, Berman SR, Kupfer DJ (1989). The Pittsburgh Sleep Quality Index: a new instrument for psychiatric practice and research. Psychiatry Res.

[66] Akman T, Yavuzsen T, Sevgen Z, Ellidokuz H, Yilmaz AU (2015). Evaluation of sleep disorders in cancer patients based on Pittsburgh Sleep Quality Index. Eur J Cancer Care (Engl).

[67] Yu M, Tatalovich Z, Gibson JT, Cronin KA (2014). Using a composite index of socioeconomic status to investigate health disparities while protecting the confidentiality of cancer registry data. Cancer Causes Control.

[68] Dieli-Conwright CM, Mortimer JE, Schroeder ET (2014). Randomized controlled trial to evaluate the effects of combined progressive exercise on metabolic syndrome in breast cancer survivors: rationale, design, and methods. BMC Cancer.

[69] Lancaster GA, Dodd S, Williamson PR (2004). Design and analysis of pilot studies: recommendations for good practice. J Eval Clin Pract.

[70] Sim J, Lewis M (2012). The size of a pilot study for a clinical trial should be calculated in relation to considerations of precision and efficiency. J Clin Epidemiol.

[71] Julious SA (2005). Sample size of 12 per group rule of thumb for a pilot study. Pharm Stat.

[72] Furmaniak AC, Menig M, Markes MH (2016). Exercise for women receiving adjuvant therapy for breast cancer. Cochrane Database Syst Rev.

[73] Boyne P, Dunning K, Carl D (2016). High-intensity interval training and moderate-intensity continuous training in ambulatory chronic stroke: feasibility study. Phys Ther.

[74] Coetsee C, Terblanche E (2017). Cerebral oxygenation during cortical activation: the differential influence of three exercise training modalities. a randomized controlled trial. Eur J Appl Physiol.

[75] Robinson MM, Lowe VJ, Nair KS (2018). Increased brain glucose uptake after 12 weeks of aerobic high-intensity interval training in young and older adults. J Clin Endocrinol Metab.

[76] Mekari S, Earle M, Martins R (2020). Effect of high intensity interval training compared to continuous training on cognitive performance in young healthy adults: a pilot study. Brain Sci.

[77] Northey JM, Pumpa KL, Quinlan C (2019). Cognition in breast cancer survivors: a pilot study of interval and continuous exercise. J Sci Med Sport.

[78] Roberts RE, Anderson EJ, Husain M (2013). White matter microstructure and cognitive function. Neuroscientist.

[79] Heisz JJ, Tejada MGM, Paolucci EM, Muir C (2016). Enjoyment for high-intensity interval exercise increases during the first six weeks of training: implications for promoting exercise adherence in sedentary adults. PLoS ONE.

[80] Ungar N, Wiskemann J, Sieverding M (2016). Physical activity enjoyment and self-efficacy as predictors of cancer patients’ physical activity level. Front Psychol.

[81] Valenzuela T, Okubo Y, Woodbury A, Lord SR, Delbaere K (2018). Adherence to technology-based exercise programs in older adults: a systematic review. J Geriatr Phys Ther.

[82] Gehring K, Kloek CJ, Aaronson NK (2018). Feasibility of a home-based exercise intervention with remote guidance for patients with stable grade II and III gliomas: a pilot randomized controlled trial. Clin Rehabil.

[83] Moore D, Jung M, Hillman CH, Kang M, Loprinzi PD (2022). Interrelationships between exercise, functional connectivity, and cognition among healthy adults: a systematic review. Psychophysiology.

[84] Bigliassi M, Cabral DF, Evans AC (2026). Improving brain health via the central executive network. J Physiol (Lond).

[85] Augusto-Oliveira M, Arrifano GP, Leal-Nazaré CG (2023). Exercise reshapes the brain: molecular, cellular, and structural changes associated with cognitive improvements. Mol Neurobiol.

[86] Al Dahhan NZ, Cox E, Nieman BJ, Mabbott DJ (2022). Cross-translational models of late-onset cognitive sequelae and their treatment in pediatric brain tumor survivors. Neuron.

[87] Derks J, Kulik S, Wesseling P (2019). Understanding cognitive functioning in glioma patients: the relevance of IDH-mutation status and functional connectivity. Brain Behav.

[88] Greutter L, Schnitzenlehner L, Klotz S (2025). Alzheimer’s disease neuropathological change in younger individuals with IDH-mutant glioma. Neurooncol Adv.

[89] Gurney JG, Kadan-Lottick NS, Packer RJ (2003). Endocrine and cardiovascular late effects among adult survivors of childhood brain tumors: childhood cancer survivor study. Cancer.

[90] Castellino SM, Ullrich NJ, Whelen MJ, Lange BJ (2014). Developing interventions for cancer-related cognitive dysfunction in childhood cancer survivors. J Natl Cancer Ins.

[91] Sharma B, Allison D, Tucker P, Mabbott D, Timmons BW (2021). Exercise trials in pediatric brain tumor: a systematic review of randomized studies. J Pediatr Hematol Oncol.

[92] Delaney A, Howell CR, Krull KR (2021). Progression of frailty in survivors of childhood cancer: a St. Jude lifetime cohort report. J Natl Cancer Inst.

[93] Piscione PJ, Bouffet E, Timmons B (2017). Exercise training improves physical function and fitness in long-term paediatric brain tumour survivors treated with cranial irradiation. Eur J Cancer.

[94] Ness KK, Krull KR, Jones KE (2013). Physiologic frailty as a sign of accelerated aging among adult survivors of childhood cancer: a report from the St Jude lifetime cohort study. J Clin Oncol.

[95] Brinkman TM, Ness KK, Li Z (2018). Attainment of functional and social independence in adult survivors of pediatric CNS tumors: a report from the St Jude lifetime cohort study. J Clin Oncol.

[96] Schulte F, Brinkman TM, Li C (2018). Social adjustment in adolescent survivors of pediatric central nervous system tumors: a report from the childhood cancer survivor study. Cancer.

[97] Moyer KH, Willard VW, Gross AM (2012). The impact of attention on social functioning in survivors of pediatric acute lymphoblastic leukemia and brain tumors. Pediatr Blood Cancer.

[98] Willard VW (2018). Social skills interventions for survivors of pediatric brain tumors: a review and reformulation. Pediatr Blood Cancer.

[99] Borrega-Mouquinho Y, Sánchez-Gómez J, Fuentes-García JP, Collado-Mateo D, Villafaina S (2021). Effects of High-intensity interval training and moderate-intensity training on stress, depression, anxiety, and resilience in healthy adults during coronavirus disease 2019 confinement: a randomized controlled trial. Front Psychol.

[100] Farah MJ, Shera DM, Savage JH (2006). Childhood poverty: specific associations with neurocognitive development. Brain Res.

[101] Johnson SB, Riis JL, Noble KG (2016). State of the art review: poverty and the developing brain. Pediatrics.

[102] Balfour JL, Kaplan GA (2002). Neighborhood environment and loss of physical function in older adults: evidence from the Alameda County study. Am J Epidemiol.

